# Efficacy and safety of a submucosal injection solution of sodium alginate for endoscopic resection in a porcine model

**DOI:** 10.1038/s41598-024-55226-y

**Published:** 2024-02-26

**Authors:** Kyung Uk Jung, Yeon Jae Lee, Jae-Young Jang, Joo Young Cho

**Affiliations:** 1 Department of Surgery, Kangbuk Samsung Hospital, Sungkyunkwan University School of Medicine, 29 Saemunan-ro, Jongno-gu, Seoul, 03181 Republic of Korea; 2 Research and Development Center of Mcnulty Pharma Co., Ltd., Seoul, Republic of Korea; 3 Departments of Gastroenterology, Department of Internal Medicine, College of Medicine, Kyung Hee University, Seoul, Republic of Korea; 4 Departments of Gastroenterology, Cha Gangnam Medical Center, Cha University College of Medicine, Seoul, Republic of Korea

**Keywords:** Gastrointestinal cancer, Gastroenterology, Oesophagogastroscopy

## Abstract

Endoscopic resection techniques require the use of submucosal injection. Normal saline and sodium hyaluronate solutions are mainly used for this purpose, but an ideal solution has not yet been developed. The aim of this study was to assess a new solution, MC-003—a novel submucosal injection solution developed with sodium alginate as the main ingredient. Normal saline, a commercial sodium hyaluronate solution (Endo-Ease), and MC-003 were examined. A total of 18 gastric submucosal cushions were created in the stomachs of six pigs. The height of mucosal elevation was measured sequentially using endoscopic sonography. After euthanizing the animals either 2 h or 5 days after the procedure, pathologic examination was performed for each injection site. Although not statistically significant over the entire study period, MC-003 showed a superior result to normal saline and an equivalent result to Endo-Ease in the submucosal cushion height and its rate of decrease. There were no adverse outcomes after injection of the three solutions and there was no pathologically identified detrimental change in the resected specimens. MC-003 creates a sufficient submucosal fluid cushion without apparent tissue damage. It can be considered as an effective submucosal injection material.

## Introduction

Endoscopic resection of gastrointestinal neoplasms has opened new horizons for cancer prevention and early treatment. Removal of precursor lesions through prophylactic screening in the asymptomatic population has contributed to a reduction in cancer incidence, and removal of early lesions has enabled successful treatment without laparotomy, which entails enormous trauma to the body. However, endoscopic procedures such as endoscopic mucosal resection (EMR) and endoscopic submucosal dissection (ESD) are accompanied by a non-negligible rate of adverse effects.

Perforation, caused by unintended full-thickness resection or delayed necrosis of remnant tissue due to thermal injury, risks the possibility of progression to peritonitis. Fragmented resection makes a definitive diagnosis difficult and there is even the possibility of a poor prognosis due to tumor spillage.

In order to facilitate the removal of lesions and prevent these problems during EMR or ESD, it is essential to maintain adequate mucosal elevation of lesions and their surroundings.

Therefore, during the EMR or ESD procedure, in order to facilitate the removal of the lesion and to prevent adverse effects such as perforation, it is important to maintain sufficient mucosal elevation for lesions and their surroundings. To promote mucosal elevation, various submucosal injection solutions have been studied in addition to normal saline^1–8^.

The aim of this study was to evaluate the efficacy and safety of MC-003, a novel submucosal injection solution for endoscopic resection developed with sodium alginate as the main ingredient, compared with normal saline and sodium hyaluronate solution.

## Materials and methods

### Subjective and measurements

**Figure 1:**
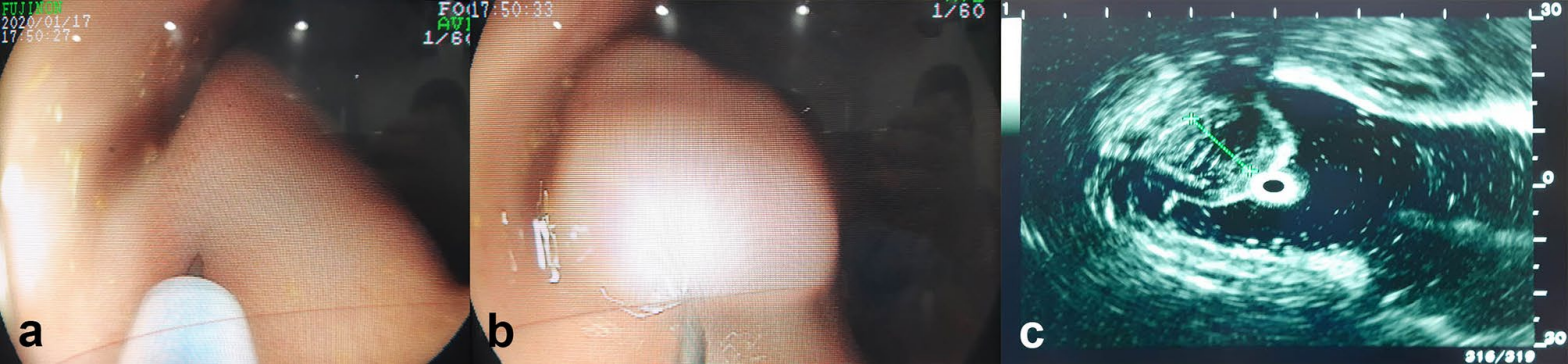
Making submucosal fluid cushions and measuring the height of the cushion by endoscopic ultrasonography. (**a**) Pre-injection. (**b**) Post-injection. (**c**) Ultrasonic view of submucosal fluid cushion.

Three solutions were examined—normal saline, Endo-Ease (UNIMED Pharm. Inc., Seoul, Korea), and MC-003 (Mcnulty Pharma Co., Ltd., Seoul, Korea). All solutions were stored and prepared at room temperature. Six male pigs between five and six months old (weighing 50–60 kg) were prepared for the study. They were anesthetized with xylazine, tiletamine, zolazepam, and isoflurane. Mechanical ventilation of the prone animals was provided during anesthesia after insertion of an endotracheal tube, and vital signs were monitored. All endoscopic procedures were performed by an experienced board certified endoscopist. Using a 23-gauge needle through the accessory channel of the gastroscope (XL-4400, Fujinon, Saitama, Japan), 5 mL of normal saline, Endo-Ease, or MC-003 were injected into the gastric submucosal layer at a separate site (minimal distance of 2 cm) in each pig, respectively. After making 18 submucosal fluid cushions in total, ultrasonography was performed by the same endoscopist at 5, 10, 15, 20, 25, 30, 35, and 40 min-time points after injection. The height of each submucosal fluid cushion was measured using an SP-900 ultrasound device (Fujifilm, Japan) (Fig. 1). Anesthesia was maintained until the last ultrasound examination was completed (40 min after submucosal injection). The pigs were euthanized after a specified period for histopathologic examination; they were euthanized using suxamethonium while under anesthesia induced with a mixture of tiletamine and zolazepam. Three of the six pigs were euthanized 2 h after the submucosal injection to assess immediate histological changes. The rest were euthanized 5 days after the procedure to examine delayed histological changes. Abdominal radiographs of each pig were obtained before and after submucosal injection and before euthanasia. Laboratory blood tests for inflammatory markers and blood chemistry were performed prior to submucosal injection and euthanasia. After euthanasia, the submucosal injection site in the stomach wall was identified and assessed histologically. The tissues were fixed in 10% formalin for more than 24 h, embedded in paraffin, sectioned into 4 μm thickness sections, and stained with hematoxylin and eosin and Masson’s Trichrome reagent. The pathologist reviewed tissue edema, congestion, inflammation, and hemorrhage around the injection site, and any significant changes were recorded based on a defined grading scale ranging from grade 0 (no specific change) to grade 1 (minimal), grade 2 (slight), grade 3 (moderate), and grade 4 (severe).

The Institutional Animal Care and Use Committee of the institution (CRONEX-IACUC: 202001007) approved this study. All procedures involving experimental animals were conducted in compliance with the principles of good laboratory animal care. This study was reported in accordance with ARRIVE guidelines.

### Statistical analysis

Continuous variables were expressed as mean ± standard deviation (1st and 3rd interquartile range). Continuous variables were all non-parametric and were calculated using the Kruskal–Wallis test. When the result of the Kruskal–Wallis test was statistically significant, a post-hoc test was performed using the Mann–Whitney U test to confirm the difference between groups. All statistical analyses was performed with SPSS Version 22 (IBM Corp., Armonk, NY, USA). *P*-values less than 0.05 were considered statistically significant.

## Results

### Chronologic changes of submucosal cushion height

**Figure 2:**
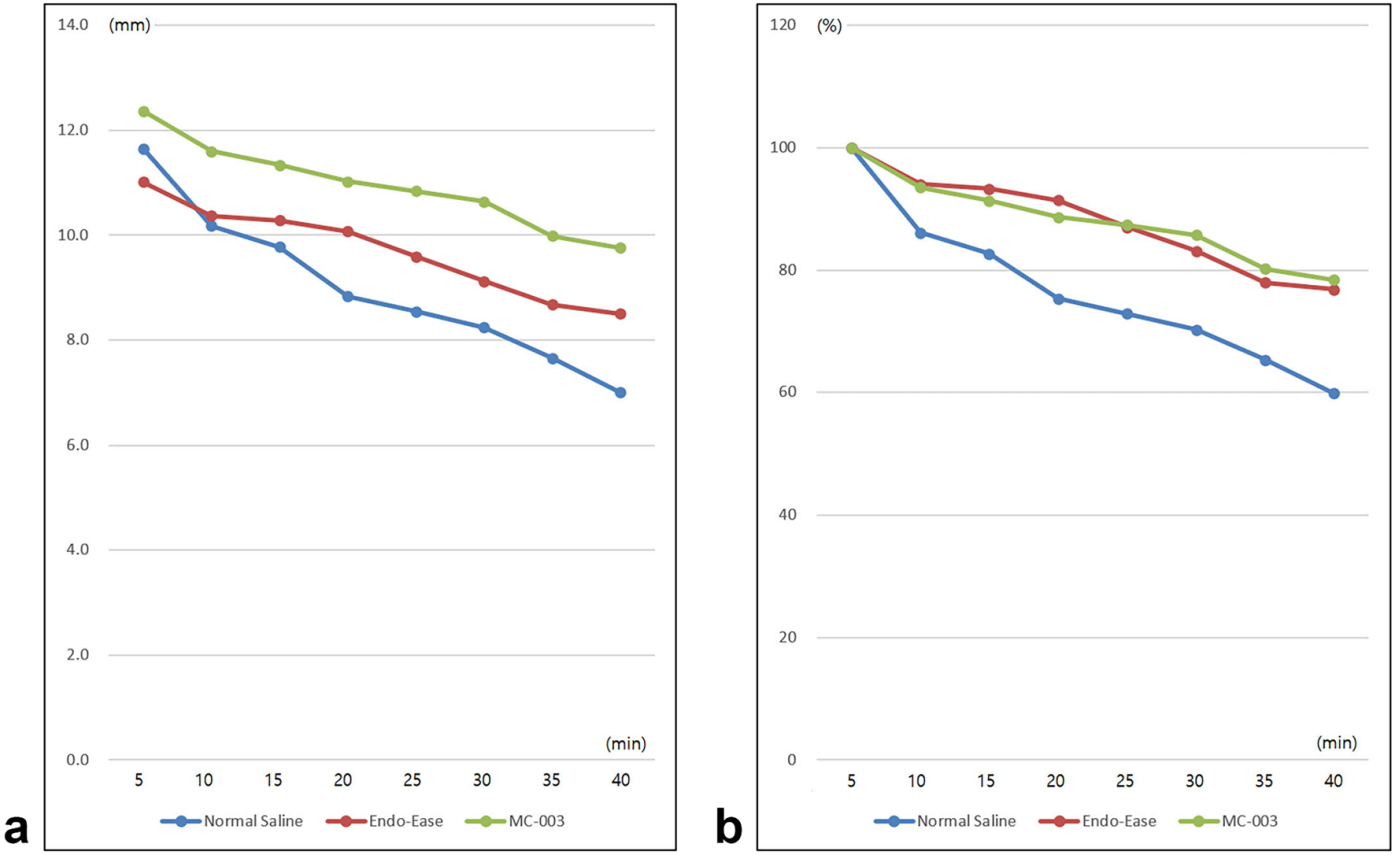
Chronologic changes in the submucosal cushion heights after injection. (**a**) Cushion heights in mm. (**b**) Cushion height ratio compared with the reference point (height at 5 min after injection).

The height of the submucosal fluid cushion decreased with time in all three substance groups. The height of the submucosal cushion was highest in the MC-003 group and lowest in the normal saline group at all time points. However, this difference was statistically significant only in measurements taken at 40 min after the procedure (Table 1, Fig. 2a).

**Table 1:** Chronologic changes of submucosal cushion heights measured by endoscopic sonography.

Time (min)	Normal Saline	Endo-Ease	MC-003	p-value
5	11.7 ± 2.1	11.0 ± 2.0	12.4 ± 1.2	NS
	(10.1–13.3)	(9.2–12.8)	(12.1–13.2)	
10	10.2 ± 2.9	10.4 ± 2.0	11.6 ± 1.5	NS
	(8.5–12.1)	(8.8–11.9)	(11.4–12.0)	
15	9.8 ± 2.8	10.3 ± 2.0	11.3 ± 1.6	NS
	(8.1–11.7)	(8.7–12.0)	(11.0–11.8)	
20	8.8 ± 2.5	10.1 ± 1.9	11.0 ± 1.7	NS
	(7.2–10.9)	(8.5–11.6)	(10.5–11.6)	
25	8.5 ± 2.3	9.6 ± 1.9	10.8 ± 1.5	NS
	(6.9–10.2)	(8.2–10.9)	(10.9–11.5)	
30	8.2 ± 2.3	9.1 ± 1.6	10.6 ± 1.6	NS
	(6.4–9.9)	(8.0–10.4)	(10.9–11.4)	
35	7.7 ± 2.1	8.7 ± 2.1	10.0 ± 2.0	NS
	(6.1–9.7)	(7.2–10.4)	(9.9–11.3)	
40	7.0 ± 2.4	8.5 ± 2.0	9.8 ± 1.9	0.044
	(5.0–9.2)	(7.1–9.9)	(10.1–10.9)	

NS, not significant.

The rate of decrease in cushion height, which was inferred from the height percentage at each time point, using the cushion height 5 min after the procedure (the first ultrasonography measurement) as a reference, was remarkably faster in the normal saline group compared with the other two groups. The decreasing rates of the Endo-Ease and MC-003 groups were similar. While this trend is clearly visible in the graph (Fig. 2b), statistical significance was only found at the 20-min time point (Table 2).

**Table 2:** Chronologic changes in submucosal cushion height ratio based on the initial measurements.

Time (min)	Normal saline	Endo-Ease	MC-003	p-value
5	100 (reference)	100 (reference)	100 (reference)	NS
10	86.1 ± 14.9	94.0 ± 4.8	93.5 ± 4.5	NS
	(86.3–95.7)	(92.7–97.0)	(90.4–96.4)	
15	82.7 ± 14.4	93.2 ± 6.4	91.3 ± 4.8	NS
	(82.0–92.2)	(87.6–98.4)	(88.5–94.1)	
20	75.3 ± 14.4	91.4 ± 4.5	88.7 ± 5.9	0.042
	(64.2–84.6)	(89.5–94.6)	(86.1–91.8)	
25	72.9 ± 12.4	87.0 ± 5.9	87.4 ± 5.9	NS
	(61.8–82.3)	(83.8–91.5)	(86.3–90.4)	
30	70.3 ± 11.8	83.1 ± 6.0	85.8 ± 9.1	NS
	(60.7–80.0)	(77.8–87.4)	(81.1–92.6)	
35	65.3 ± 12.1	77.9 ± 8.7	80.2 ± 12.4	NS
	(55.2–72.0)	(72.0–84.8)	(76.5–90.1)	
40	59.9 ± 16.3	76.8 ± 9.3	78.4 ± 11.9	NS
	(46.0–73.0)	(68.5–83.4)	(74.2–86.6)	

NS, not significant.

### Systemic effects of submucosal injection

All animals survived after the procedure until sacrifice (half for 2 h, and the other half for 5 days after the procedure). None of the subjects showed abnormal clinical signs until euthanasia. In the radiographs taken before and after the submucosal fluid injection, no specific findings were found except for an increase in intestinal gas after the procedure. There was no change in the hematological and serum biochemical tests except for an insignificant increase in the white blood cell count and neutrophils and eosinophils after the procedure.

### Histologic changes of the submucosal injection site

**Figure 3:**
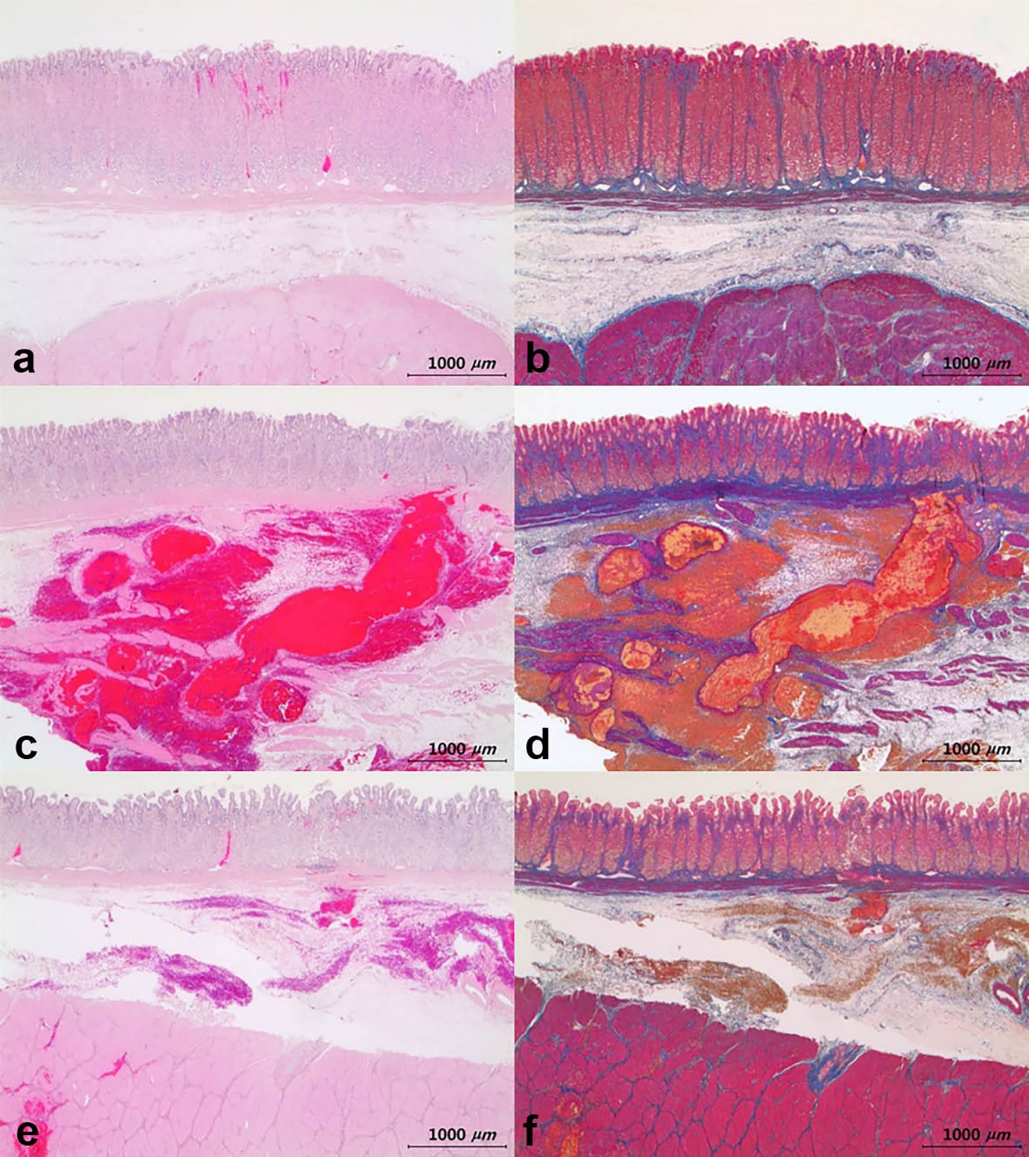
Histopathology of submucosal injection site obtained from a pig euthanized on the day of the procedure. (**a**, **b**) Tissue from the normal saline group. Hemorrhage in the mucosa. Hematoxylin and Eosin, and Masson’s Trichrome stained, respectively. (**c**, **d**) Tissue from the Endo-Ease group. Hemorrhage in the submucosa. Hematoxylin and Eosin, and Masson’s Trichrome stained, respectively. (**e**, **f**) Tissue from the MC-003 group. Hemorrhage in mucosa, submucosa, and muscularis propria. Hematoxylin and Eosin, and Masson’s Trichrome stained, respectively.

**Figure 4:**
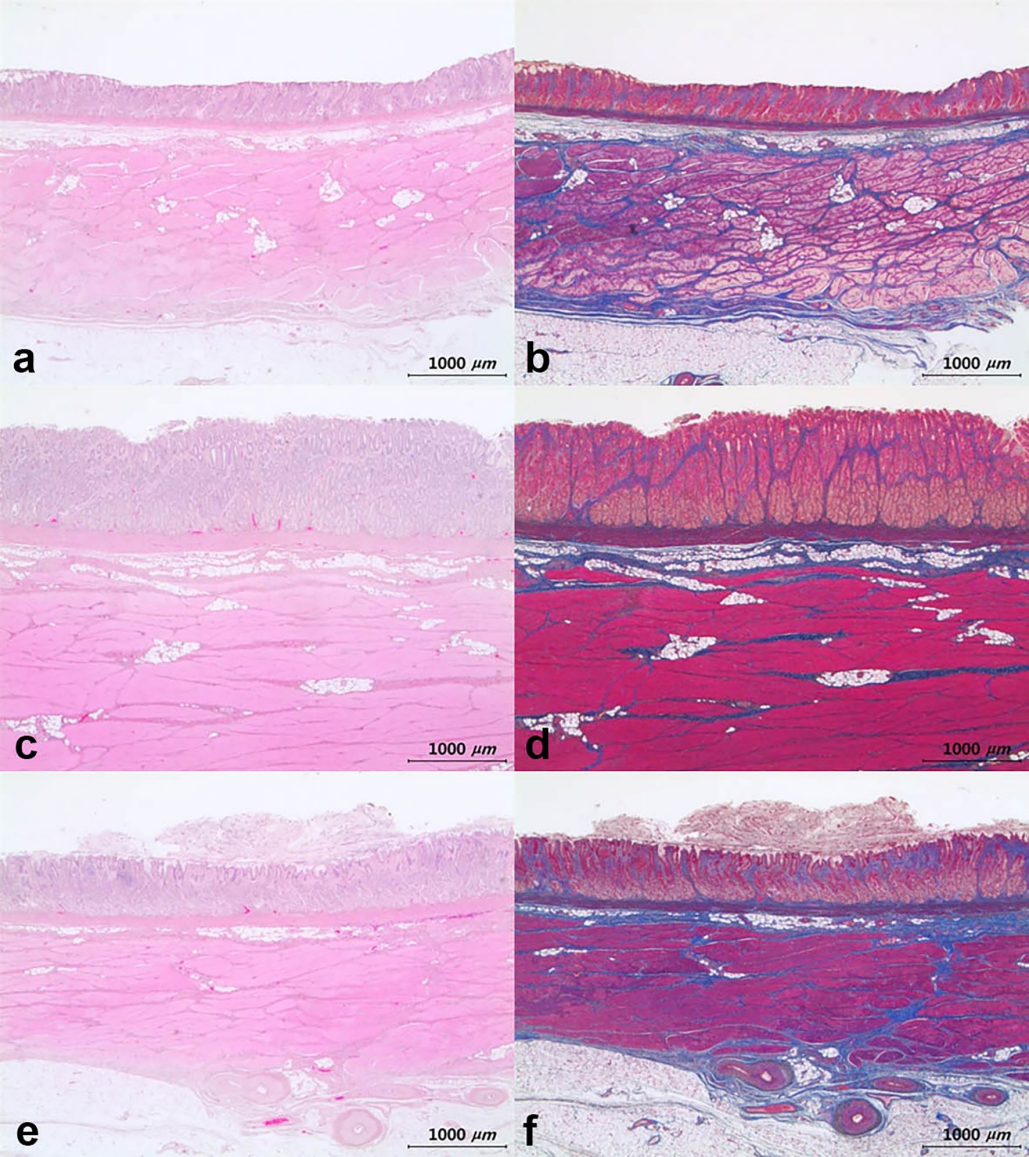
Histopathology of submucosal injection site obtained from the pig euthanized 5 days after the procedure. (**a**, **b**) Tissue from the normal saline group. No significant lesions. Hematoxylin and Eosin, and Masson’s Trichrome stained, respectively. (**c**, **d**) Tissue from the Endo-Ease group. Congestion in the mucosa. Hematoxylin and Eosin, and Masson’s Trichrome stained, respectively. (**e**, **f**) Tissue from the MC-003 group. No significant lesions. Hematoxylin and Eosin, and Masson’s Trichrome stained, respectively.

In the tissues obtained from the three pigs euthanized on the day of the procedure, mucosal or submucosal hemorrhage was observed in all tissues except one belonging to the normal saline group (Fig. 3). In the tissues obtained from euthanized animals 5 days after the procedure, hyperemia of the gastric mucosal layer was observed in one tissue in the normal saline group and two in the Endo-Ease group (Fig. 4). Table 3 displays histopathological changes observed at the injection site in each animal. There were no other specific histologic changes, such as hemorrhage beyond the submucosal layer, inflammation, and perforation.

**Table 3:** Histopathology of submucosal injection site.

Material	Days	Animal ID	Congestion	Hemorrhage
Normal Saline	0 (2 h)	1	0	1
		2	0	1
		3	0	0
	5	4	0	0
		5	0	0
		6	1	0
Endo-Ease	0 (2 h)	1	0	3
		2	0	3
		3	0	2
	5	4	0	0
		5	1	0
		6	1	0
MC-003	0 (2 h)	1	0	1
		2	0	1
		3	0	3
	5	4	0	0
		5	0	0
		6	0	0

0, no significant lesions; 1, minimal; 2, slight; 3, moderate; 4, severe.

## Discussion

The ideal submucosal injection solution should have the following characteristics: (1) it should provide a thick submucosal fluid cushion and remain in the submucosal space long enough to allow completion of the endoscopic procedure; (2) it should not cause severe hemorrhage or local inflammatory reaction and should be absorbed and disappear from the site of injection after a reasonable time when the procedure has been completed; (3) it should not distort the tissue but rather, enable precise pathologic staging from the acquired tissue; (4) it should not have a detrimental systemic effect when absorbed through the gastrointestinal tract; (5) it should be convenient to use, easy to store and transport, and be reasonably priced.

Because it is readily available, inexpensive, and causes very little tissue damage due to its isotonic nature, normal saline is most frequently employed to produce a submucosal cushion for endoscopic procedures^9^. However, owing to its low viscosity and high absorbance into tissues, it has the disadvantage of requiring multiple injections to maintain mucosal elevation during the procedure^9,10^. If adequate mucosal elevation is not maintained, the risk of adverse effects such as perforation increases, and the procedure time is prolonged during multiple injections, which is burdensome for the patient.

High osmotic submucosal injection solutions such as glucose solution, fibrinogen combination solution, or sodium hyaluronate, have been developed to sustain longer mucosal elevation compared with normal saline. However, the chance of tissue injury increases as the concentration of glucose solution rises, and there is a risk of virus infection in using a fibrinogen mixture purified from human serum^2,11–13^. Although sodium hyaluronate has been reported to be the most effective submucosal injection for creating and maintaining mucosal elevation and has been successfully commercialized^9,10,14,15^, it also has the disadvantage of high cost.

Sodium alginate is one of the polysaccharides contained in the kelp, *Phaeophyceae*. It is a linear copolymer with homopolymeric blocks of (1–4)-linked beta-D-mannuronate and its C-5 epimer, alpha-l-guluronate (G) residues, respectively covalently linked in different sequences or blocks. It has the potential to be a new submucosal injection material for use in endoscopic procedures due to its viscosity, protective effect on mucous membranes, and hemostatic effect by accelerating fibrin formation^16,17^. Moreover, the cost is very low, less than 1/100^th^ that of sodium hyaluronate at the same dosage^18^. Furthermore, it is nontoxic, nonallergenic, readily obtainable in bulk, and can be stored at room temperature. Since Eun et al. first demonstrated a 1% solution of sodium alginate as a sufficient submucosal injection solution for endoscopic procedures with results of ex vivo porcine and in vivo canine animal experiments in 2007^19^, there have been several reports examining sodium alginate-based solutions for submucosal injection^18,20,21^. Akagi et al. showed that submucosal injection of 3% sodium alginate is a reliable method^22^. They also argued that it should be more diluted for commercial use, reporting discomfort during injection and inhomogenous elevation of the tissue due to the high viscosity of the solution^18^. More recently, Kusano et al. and Uemura et al. examined a 0.6% sodium alginate solution^20,21^. At this concentration, sodium alginate demonstrated non-inferiority against a 0.4% sodium hyaluronate solution, with a higher efficacy rate of submucosal injection during ESD (91.7% vs. 88.7% in the sodium alginate and sodium hyaluronate groups, respectively)^21^. However, the optimal composition of sodium alginate solution for widespread use in actual clinical practice is still being investigated.

The biomaterial graft MC-003 is a novel submucosal injection solution developed with sodium alginate as the main ingredient. This product is a low-viscosity solution before injection and is made at a concentration that can easily pass through an endoscopic needle. When it is injected into the submucosal layer, sodium ions of sodium alginate are substituted with potassium ions from body fluid to form a gel. Indigo carmine is added to the initial colorless and translucent fluid to increase the visibility of the lesion and make the procedure easier^22^.

In this study, the novel submucosal injection solution MC-003 was assessed in comparison with normal saline-indigo carmine mixed solution and a commercially licensed 0.4% sodium hyaluronate solution, Endo-Ease. Normal saline-indigo carmine-mixed solution is widely used as a standard material for mucosal elevation during EMR or ESD and has been used as a control to evaluate the safety and efficacy of experimental injection solutions^9,14,15^. Endo-Ease was selected as a representative product of 0.4% sodium hyaluronate, the only commercially successful solution among various solutions examined so far. Pigs were selected as experimental animals because their digestive structures are histologically and functionally similar to those of humans^23^. Although it did not show statistical significance over the entire study period, MC-003 showed a superior result to that of normal saline and an equivalent result to that of Endo-Ease in the submucosal cushion height and rate of decrease. No adverse outcomes after injection of the three solutions were observed and there were no pathologically identified detrimental changes in the resected specimens.

This is a pilot study that investigates the potential of MC-003 as a submucosal injection solution during endoscopic procedures. The number of subjects is small and only submucosal injection was performed. Actual tissue resection procedures such as EMR or ESD were not carried out. The study results were limited to observing the characteristics of the submucosal cushion after submucosal injection, chronologic changes of submucosal cushion height, and tissue changes in pathological examination. Nevertheless, the results of this study illustrate the safety and future potential of MC-003. Considering the low cost of sodium alginate, MC-003 may be a promising alternative to current sodium hyaluronate solutions.

MC-003, a novel submucosal injection solution developed with sodium alginate showed sufficient mucosal initial lifting and an effect equivalent to that of a sodium hyaluronate-based solution in an in vivo porcine model. Although large scale human studies are needed in the future, our results may expand the submucosal injection solution options available in clinical practice.

## Data Availability

The datasets generated during and/or analysed during the current study are available from the corresponding author on reasonable request.
